# (1S*,4′S*,5R*)-1-Isobutyl-5-meth­oxy-2′,3-dimethyl-4,6-dioxa-2-aza­spiro­[bicyclo­[3.2.0]hept-2-ene-7,4′-isoquinoline]-1′,3′(2′*H*,4′*H*)-dione

**DOI:** 10.1107/S1600536811015327

**Published:** 2011-04-29

**Authors:** Hoong-Kun Fun, Ching Kheng Quah, Chengmei Huang, Haitao Yu

**Affiliations:** a X-ray Crystallography Unit, School of Physics, Universiti Sains Malaysia, 11800 USM, Penang, Malaysia; b School of Chemistry and Chemical Engineering, Nanjing University, Nanjing, 210093, People’s Republic of China

## Abstract

In the isoquinoline ring system of the title compound, C_19_H_22_N_2_O_5_, the N-heterocyclic ring is in a half-chair conformation. The dioxa-2-aza­spiro ring is essentially planar [maximum deviation of 0.025 (1) Å] and forms a dihedral angle of 23.51 (5)° with the benzene ring. In the crystal, mol­ecules are linked *via* weak inter­molecular C—H⋯O and C—H⋯N hydrogen bonds into chains along [010].

## Related literature

For general background to and the potential biological activity of the title compound, see: Pollers-Wieers *et al.* (1981 ▶); Malamas *et al.* (1994 ▶); Yu *et al.* (2010 ▶); Du *et al.* (2008 ▶); Chen *et al.* (2006 ▶); Zhang *et al.* (2006 ▶); Mitchell *et al.* (1995 ▶, 2000 ▶); Harris *et al.* (2005 ▶); Wang *et al.* (2010 ▶); Huang *et al.* (2011 ▶). For the stability of the temperature controller used in the data collection, see: Cosier & Glazer (1986 ▶). For standard bond-length data, see: Allen *et al.* (1987 ▶). For ring conformations, see: Cremer & Pople (1975 ▶). For a related structure, see: Fun *et al.* (2011 ▶). 
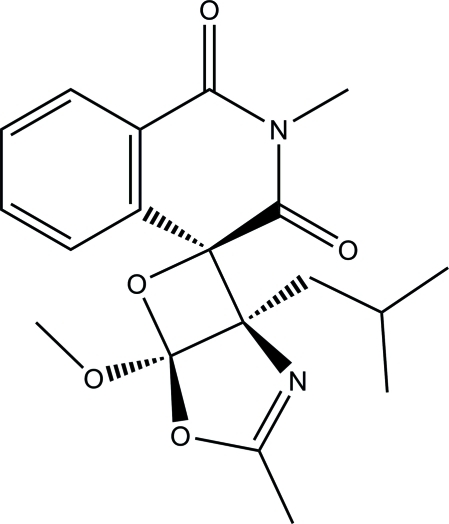


## Experimental

### 

#### Crystal data

C_19_H_22_N_2_O_5_
*M* *_r_* = 358.39
Monoclinic, 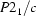

*a* = 8.0488 (1) Å
*b* = 13.6065 (2) Å
*c* = 16.7880 (2) Å
β = 106.298 (1)°
*V* = 1764.67 (4) Å^3^
*Z* = 4
Mo *K*α radiation
μ = 0.10 mm^−1^
*T* = 100 K
0.25 × 0.16 × 0.12 mm

#### Data collection

Bruker SMART APEXII CCD area-detector diffractometer
Absorption correction: multi-scan (*SADABS*; Bruker, 2009 ▶) *T*_min_ = 0.976, *T*_max_ = 0.988
24267 measured reflections
5139 independent reflections
4476 reflections with *I* > 2σ(*I*)
*R*_int_ = 0.025

#### Refinement

*R*[*F* ^2^ > 2σ(*F* ^2^)] = 0.038
*wR*(*F* ^2^) = 0.103
*S* = 1.03
5139 reflections
240 parameters
H-atom parameters constrained
Δρ_max_ = 0.44 e Å^−3^
Δρ_min_ = −0.24 e Å^−3^

### 

Data collection: *APEX2* (Bruker, 2009 ▶); cell refinement: *SAINT* (Bruker, 2009 ▶); data reduction: *SAINT*; program(s) used to solve structure: *SHELXTL* (Sheldrick, 2008 ▶); program(s) used to refine structure: *SHELXTL*; molecular graphics: *SHELXTL*; software used to prepare material for publication: *SHELXTL* and *PLATON* (Spek, 2009 ▶).

## Supplementary Material

Crystal structure: contains datablocks global, I. DOI: 10.1107/S1600536811015327/lh5238sup1.cif

Structure factors: contains datablocks I. DOI: 10.1107/S1600536811015327/lh5238Isup2.hkl

Additional supplementary materials:  crystallographic information; 3D view; checkCIF report

## Figures and Tables

**Table 1:** Hydrogen-bond geometry (Å, °)

*D*—H⋯*A*	*D*—H	H⋯*A*	*D*⋯*A*	*D*—H⋯*A*
C16—H16*B*⋯O5^i^	0.96	2.57	3.4213 (14)	148
C17—H17*C*⋯N2^ii^	0.96	2.59	3.5119 (14)	162

Symmetry codes: (i) 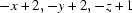; (ii) 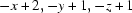.

**Fig. 1.**
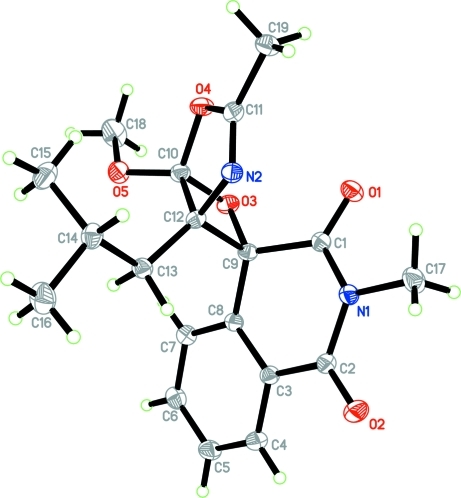
The molecular structure of the title compound showing 50% probability displacement ellipsoids for non-H atoms.

**Fig. 2.**
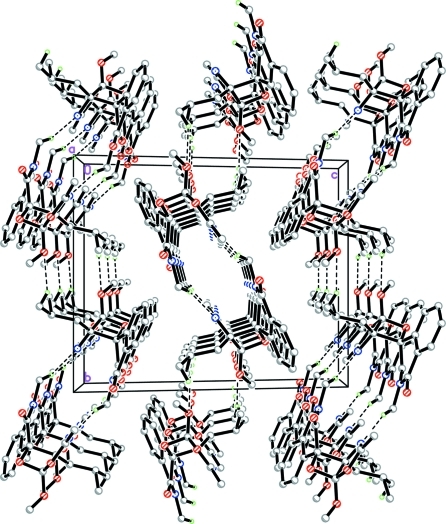
Part of the crystal structure of the title compound, viewed along the a axis. H atoms not involved in hydrogen bonds (dashed lines) have been omitted for clarity.

## supplementary crystallographic information

### Comment

Oxazole rings are found in some bioactive natural products such as Annuloline and Ostreogrycin A. Compounds with oxazole moiety have been found to have inhibition activity on malignant tumors (Harris *et al.*, 2005). Additionally, many natural products especially the alkaloids containing the isoquinoline or oxazole ring are bioactive. As such there has been intense development of methodology to construct such moieties (Wang *et al.*, 2010). Isoquinolines are often found in bioactive natural products. They have been used to build blocks of benzo[c]phenanthridine alkaloids (Pollers-Wieers *et al.*, 1981; Malamas *et al.*, 1994; Yu *et al.*, 2010). Isoquinoline-1,3,4-trione derivatives were reported to be a kind of small molecular inhibitor against caspase-3 which can promote apoptosis of the cells. (Du *et al.*, 2008; Chen *et al.*, 2006). They can also attenuate apoptosis of neuronal cells induced by β-amyloid.(Zhang *et al.*, 2006). Isoquinoline-1,3,4-trione and its derivatives have been reported to be redox mediators of photosystems I and have been used as herbicides (Mitchell *et al.*, 2000; 1995). The title compound which was derived from isoquinoline-1,3,4-trione and oxazoles (Huang *et al.*, 2011) may have potential use in biochemical and pharmaceutical fields. We report herein the crystal structure of the title compound with a relative configuration of (1S^*^, 4'S^*^, 5R^*^).

In the title racemic compound, Fig. 1, atoms C9, C10 and C12 are the chiral centers. The isoquinoline ring system (N1/C1-C9) is not completely planar, the *N*-heterocyclic ring (N1/C1-C3/C8/C9) being distorted towards a half-chair conformation with atoms N1/C2/C3/C8  forming the best least-squares plane and and atoms C1 and C9 are 0.2278 (8) and -0.3215 (8)Å, respectively, from this plane. The puckering parameters (Cremer & Pople, 1975) are Q = 0.3656 (10)) Å, Θ =   114.98 (16)° and φ =274.96 (16)°. The bond lengths (Allen *et al.*, 1987) and angles are within normal ranges and comparable to a related structure (Fun *et al.*, 2011). The dioxa-2-azaspiro ring (N2/O4/C10-C12) is essentially planar [maximum deviation of 0.025 (1) Å at atom O4] and is inclined at a dihedral angle of 23.51 (5)° with the benzene ring (C3-C8).

In the crystal (Fig. 2), molecules are linked *via* weak intermolecular C16–H16B···O5^i^ and C17–H17C···N2^ii^ hydrogen bonds (see Table 1 for symmetry codes) into one-dimensional chains along [010].

### Experimental

The title compound was the main product from the photoreaction between isoquinoline-1,3,4-trione and 4-isobutyl-5-methoxy-2-methyloxazole. The compound was purified by flash column chromatography with ethyl acetate/petroleum ether (1:4) as eluents. X-ray quality crystals of the title compound was obtained from slow evaporation of an acetone and petroleum ether solution of the title compund (1:5) (*m.p.* 421-423 K).

### Refinement

All H atoms were positioned geometrically and refined using a riding model with C–H = 0.93 - 0.98 Å and *U*_iso_(H) = 1.2 or 1.5 *U*_eq_(C). A rotating-group model was applied for the methyl groups.

### Crystal data

**Table:**

C_19_H_22_N_2_O_5_	*F*(000) = 760
*M**_r_* = 358.39	*D*_x_ = 1.349 Mg m^−^^3^
Monoclinic, *P*2_1_/*c*	Mo *K*α radiation, λ = 0.71073 Å
Hall symbol: -P 2ybc	Cell parameters from 9893 reflections
*a* = 8.0488 (1) Å	θ = 3.0–35.8°
*b* = 13.6065 (2) Å	µ = 0.10 mm^−^^1^
*c* = 16.7880 (2) Å	*T* = 100 K
β = 106.298 (1)°	Block, colourless
*V* = 1764.67 (4) Å^3^	0.25 × 0.16 × 0.12 mm
*Z* = 4	

### Data collection

**Table:**

Bruker SMART APEXII CCD area-detector diffractometer	5139 independent reflections
Radiation source: fine-focus sealed tube	4476 reflections with *I* > 2σ(*I*)
graphite	*R*_int_ = 0.025
φ and ω scans	θ_max_ = 30.0°, θ_min_ = 2.5°
Absorption correction: multi-scan (*SADABS*; Bruker, 2009)	*h* = −11→11
*T*_min_ = 0.976, *T*_max_ = 0.988	*k* = −19→19
24267 measured reflections	*l* = −23→23

### Refinement

**Table:**

Refinement on *F*^2^	Primary atom site location: structure-invariant direct methods
Least-squares matrix: full	Secondary atom site location: difference Fourier map
*R*[*F*^2^ > 2σ(*F*^2^)] = 0.038	Hydrogen site location: inferred from neighbouring sites
*wR*(*F*^2^) = 0.103	H-atom parameters constrained
*S* = 1.03	*w* = 1/[σ^2^(*F*_o_^2^) + (0.0553*P*)^2^ + 0.5274*P*] where *P* = (*F*_o_^2^ + 2*F*_c_^2^)/3
5139 reflections	(Δ/σ)_max_ = 0.001
240 parameters	Δρ_max_ = 0.44 e Å^−^^3^
0 restraints	Δρ_min_ = −0.24 e Å^−^^3^

### Special details

**Table:**

Experimental. The crystal was placed in the cold stream of an Oxford Cryosystems Cobra open-flow nitrogen cryostat (Cosier & Glazer, 1986) operating at 100.0 (1) K.
Geometry. All esds (except the esd in the dihedral angle between two l.s. planes) are estimated using the full covariance matrix. The cell esds are taken into account individually in the estimation of esds in distances, angles and torsion angles; correlations between esds in cell parameters are only used when they are defined by crystal symmetry. An approximate (isotropic) treatment of cell esds is used for estimating esds involving l.s. planes.
Refinement. Refinement of F^2^ against ALL reflections. The weighted R-factor wR and goodness of fit S are based on F^2^, conventional R-factors R are based on F, with F set to zero for negative F^2^. The threshold expression of F^2^ > 2sigma(F^2^) is used only for calculating R-factors(gt) etc. and is not relevant to the choice of reflections for refinement. R-factors based on F^2^ are statistically about twice as large as those based on F, and R- factors based on ALL data will be even larger.

### Fractional atomic coordinates and isotropic or equivalent isotropic displacement parameters (Å^2^)

**Table:**

	*x*	*y*	*z*	*U*_iso_*/*U*_eq_	
O1	0.79496 (9)	0.57309 (5)	0.66503 (5)	0.02050 (15)	
O2	1.34091 (9)	0.52679 (5)	0.64902 (5)	0.02368 (16)	
O3	0.82385 (8)	0.77489 (5)	0.67549 (4)	0.01707 (14)	
O4	0.57984 (8)	0.76986 (5)	0.55910 (4)	0.01918 (15)	
O5	0.74064 (9)	0.91191 (5)	0.58917 (5)	0.02068 (15)	
N1	1.05809 (10)	0.55116 (6)	0.64124 (5)	0.01658 (16)	
N2	0.75988 (10)	0.67859 (6)	0.50490 (5)	0.01618 (16)	
C1	0.92559 (11)	0.60875 (7)	0.65547 (5)	0.01528 (17)	
C2	1.22975 (11)	0.58267 (7)	0.65818 (6)	0.01617 (17)	
C3	1.26757 (11)	0.68377 (7)	0.69139 (6)	0.01539 (17)	
C4	1.44041 (12)	0.71107 (7)	0.72596 (6)	0.01982 (19)	
H4A	1.5292	0.6675	0.7255	0.024*	
C5	1.47831 (12)	0.80359 (8)	0.76086 (6)	0.0219 (2)	
H5A	1.5927	0.8212	0.7859	0.026*	
C6	1.34573 (13)	0.87042 (7)	0.75865 (6)	0.01974 (19)	
H6A	1.3722	0.9327	0.7815	0.024*	
C7	1.17372 (12)	0.84428 (7)	0.72237 (6)	0.01706 (17)	
H7A	1.0857	0.8895	0.7198	0.020*	
C8	1.13408 (11)	0.75022 (7)	0.68989 (5)	0.01429 (16)	
C9	0.95168 (11)	0.71880 (6)	0.64877 (5)	0.01391 (16)	
C10	0.74669 (11)	0.81247 (7)	0.59376 (6)	0.01633 (17)	
C11	0.60784 (12)	0.69179 (7)	0.51199 (6)	0.01745 (18)	
C12	0.86979 (11)	0.75430 (7)	0.55523 (5)	0.01432 (16)	
C13	0.99210 (11)	0.80256 (7)	0.51287 (6)	0.01624 (17)	
H13A	1.0911	0.7597	0.5187	0.019*	
H13B	1.0343	0.8632	0.5419	0.019*	
C14	0.91552 (13)	0.82618 (7)	0.42050 (6)	0.01925 (18)	
H14A	0.8691	0.7654	0.3914	0.023*	
C15	0.76970 (14)	0.90189 (8)	0.40468 (7)	0.0259 (2)	
H15A	0.6740	0.8751	0.4211	0.039*	
H15B	0.7328	0.9179	0.3467	0.039*	
H15C	0.8106	0.9602	0.4363	0.039*	
C16	1.06068 (15)	0.86338 (9)	0.38598 (7)	0.0274 (2)	
H16A	1.1485	0.8138	0.3932	0.041*	
H16B	1.1101	0.9219	0.4151	0.041*	
H16C	1.0146	0.8778	0.3280	0.041*	
C17	1.02112 (14)	0.44773 (7)	0.61770 (7)	0.0245 (2)	
H17A	0.9002	0.4403	0.5898	0.037*	
H17B	1.0509	0.4075	0.6666	0.037*	
H17C	1.0881	0.4279	0.5813	0.037*	
C18	0.62742 (16)	0.95764 (8)	0.63135 (9)	0.0323 (3)	
H18A	0.6249	1.0273	0.6220	0.048*	
H18B	0.6693	0.9447	0.6898	0.048*	
H18C	0.5127	0.9314	0.6103	0.048*	
C19	0.45239 (12)	0.63113 (8)	0.47491 (7)	0.0226 (2)	
H19A	0.4805	0.5812	0.4404	0.034*	
H19B	0.3618	0.6721	0.4421	0.034*	
H19C	0.4143	0.6007	0.5183	0.034*	

### Atomic displacement parameters (Å^2^)

**Table:**

	*U*^11^	*U*^22^	*U*^33^	*U*^12^	*U*^13^	*U*^23^
O1	0.0177 (3)	0.0217 (3)	0.0236 (3)	−0.0037 (3)	0.0082 (3)	0.0008 (3)
O2	0.0187 (3)	0.0198 (3)	0.0330 (4)	0.0042 (3)	0.0081 (3)	−0.0012 (3)
O3	0.0149 (3)	0.0201 (3)	0.0172 (3)	0.0031 (2)	0.0062 (2)	−0.0021 (2)
O4	0.0126 (3)	0.0210 (3)	0.0236 (4)	0.0003 (2)	0.0045 (2)	−0.0023 (3)
O5	0.0209 (3)	0.0146 (3)	0.0297 (4)	0.0029 (2)	0.0123 (3)	−0.0011 (3)
N1	0.0156 (3)	0.0137 (3)	0.0207 (4)	−0.0006 (3)	0.0056 (3)	−0.0008 (3)
N2	0.0156 (3)	0.0163 (4)	0.0151 (4)	−0.0011 (3)	0.0018 (3)	−0.0005 (3)
C1	0.0155 (4)	0.0165 (4)	0.0135 (4)	−0.0004 (3)	0.0034 (3)	0.0001 (3)
C2	0.0151 (4)	0.0166 (4)	0.0164 (4)	0.0010 (3)	0.0039 (3)	0.0021 (3)
C3	0.0144 (4)	0.0166 (4)	0.0149 (4)	0.0001 (3)	0.0036 (3)	0.0008 (3)
C4	0.0145 (4)	0.0223 (5)	0.0213 (4)	0.0010 (3)	0.0027 (3)	0.0022 (4)
C5	0.0163 (4)	0.0247 (5)	0.0218 (5)	−0.0041 (3)	0.0005 (3)	0.0009 (4)
C6	0.0220 (4)	0.0196 (4)	0.0166 (4)	−0.0048 (3)	0.0037 (3)	−0.0022 (3)
C7	0.0180 (4)	0.0176 (4)	0.0159 (4)	−0.0002 (3)	0.0054 (3)	−0.0013 (3)
C8	0.0138 (4)	0.0167 (4)	0.0125 (4)	−0.0005 (3)	0.0038 (3)	0.0006 (3)
C9	0.0127 (4)	0.0152 (4)	0.0147 (4)	0.0008 (3)	0.0053 (3)	−0.0012 (3)
C10	0.0135 (4)	0.0163 (4)	0.0193 (4)	0.0009 (3)	0.0050 (3)	−0.0006 (3)
C11	0.0166 (4)	0.0175 (4)	0.0167 (4)	−0.0001 (3)	0.0023 (3)	0.0015 (3)
C12	0.0133 (4)	0.0147 (4)	0.0145 (4)	0.0015 (3)	0.0033 (3)	−0.0005 (3)
C13	0.0156 (4)	0.0177 (4)	0.0157 (4)	−0.0001 (3)	0.0048 (3)	0.0008 (3)
C14	0.0237 (4)	0.0172 (4)	0.0160 (4)	−0.0003 (3)	0.0041 (3)	0.0003 (3)
C15	0.0270 (5)	0.0243 (5)	0.0229 (5)	0.0044 (4)	0.0011 (4)	0.0045 (4)
C16	0.0345 (6)	0.0300 (6)	0.0210 (5)	0.0003 (4)	0.0133 (4)	0.0043 (4)
C17	0.0248 (5)	0.0156 (4)	0.0353 (6)	−0.0031 (4)	0.0122 (4)	−0.0051 (4)
C18	0.0340 (6)	0.0195 (5)	0.0530 (8)	0.0043 (4)	0.0281 (5)	−0.0040 (5)
C19	0.0174 (4)	0.0243 (5)	0.0235 (5)	−0.0044 (3)	0.0015 (3)	0.0008 (4)

### Geometric parameters (Å, °)

**Table:**

O1—C1	1.2088 (11)	C9—C12	1.5978 (12)
O2—C2	1.2164 (11)	C10—C12	1.5447 (12)
O3—C10	1.4323 (11)	C11—C19	1.4823 (13)
O3—C9	1.4496 (10)	C12—C13	1.5162 (12)
O4—C11	1.3801 (12)	C13—C14	1.5334 (13)
O4—C10	1.4282 (11)	C13—H13A	0.9700
O5—C10	1.3554 (11)	C13—H13B	0.9700
O5—C18	1.4428 (12)	C14—C15	1.5278 (14)
N1—C1	1.3970 (11)	C14—C16	1.5288 (14)
N1—C2	1.3976 (11)	C14—H14A	0.9800
N1—C17	1.4694 (12)	C15—H15A	0.9600
N2—C11	1.2750 (12)	C15—H15B	0.9600
N2—C12	1.4623 (12)	C15—H15C	0.9600
C1—C9	1.5205 (13)	C16—H16A	0.9600
C2—C3	1.4834 (13)	C16—H16B	0.9600
C3—C4	1.3989 (12)	C16—H16C	0.9600
C3—C8	1.3990 (12)	C17—H17A	0.9600
C4—C5	1.3860 (14)	C17—H17B	0.9600
C4—H4A	0.9300	C17—H17C	0.9600
C5—C6	1.3945 (14)	C18—H18A	0.9600
C5—H5A	0.9300	C18—H18B	0.9600
C6—C7	1.3932 (13)	C18—H18C	0.9600
C6—H6A	0.9300	C19—H19A	0.9600
C7—C8	1.3926 (13)	C19—H19B	0.9600
C7—H7A	0.9300	C19—H19C	0.9600
C8—C9	1.4987 (12)		
			
C10—O3—C9	92.64 (6)	N2—C12—C10	104.34 (7)
C11—O4—C10	105.06 (7)	C13—C12—C10	123.48 (8)
C10—O5—C18	114.85 (8)	N2—C12—C9	111.75 (7)
C1—N1—C2	123.48 (8)	C13—C12—C9	116.67 (7)
C1—N1—C17	118.46 (8)	C10—C12—C9	83.08 (6)
C2—N1—C17	117.55 (8)	C12—C13—C14	115.81 (8)
C11—N2—C12	106.72 (8)	C12—C13—H13A	108.3
O1—C1—N1	122.12 (9)	C14—C13—H13A	108.3
O1—C1—C9	123.26 (8)	C12—C13—H13B	108.3
N1—C1—C9	114.32 (7)	C14—C13—H13B	108.3
O2—C2—N1	120.18 (9)	H13A—C13—H13B	107.4
O2—C2—C3	123.13 (8)	C15—C14—C16	110.02 (9)
N1—C2—C3	116.61 (8)	C15—C14—C13	112.85 (8)
C4—C3—C8	120.34 (9)	C16—C14—C13	108.64 (8)
C4—C3—C2	118.52 (8)	C15—C14—H14A	108.4
C8—C3—C2	121.13 (8)	C16—C14—H14A	108.4
C5—C4—C3	119.46 (9)	C13—C14—H14A	108.4
C5—C4—H4A	120.3	C14—C15—H15A	109.5
C3—C4—H4A	120.3	C14—C15—H15B	109.5
C4—C5—C6	120.33 (9)	H15A—C15—H15B	109.5
C4—C5—H5A	119.8	C14—C15—H15C	109.5
C6—C5—H5A	119.8	H15A—C15—H15C	109.5
C7—C6—C5	120.29 (9)	H15B—C15—H15C	109.5
C7—C6—H6A	119.9	C14—C16—H16A	109.5
C5—C6—H6A	119.9	C14—C16—H16B	109.5
C8—C7—C6	119.74 (9)	H16A—C16—H16B	109.5
C8—C7—H7A	120.1	C14—C16—H16C	109.5
C6—C7—H7A	120.1	H16A—C16—H16C	109.5
C7—C8—C3	119.77 (8)	H16B—C16—H16C	109.5
C7—C8—C9	121.99 (8)	N1—C17—H17A	109.5
C3—C8—C9	118.17 (8)	N1—C17—H17B	109.5
O3—C9—C8	113.32 (7)	H17A—C17—H17B	109.5
O3—C9—C1	111.77 (7)	N1—C17—H17C	109.5
C8—C9—C1	112.62 (7)	H17A—C17—H17C	109.5
O3—C9—C12	90.69 (6)	H17B—C17—H17C	109.5
C8—C9—C12	116.56 (7)	O5—C18—H18A	109.5
C1—C9—C12	110.06 (7)	O5—C18—H18B	109.5
O5—C10—O4	111.56 (7)	H18A—C18—H18B	109.5
O5—C10—O3	114.19 (8)	O5—C18—H18C	109.5
O4—C10—O3	110.53 (7)	H18A—C18—H18C	109.5
O5—C10—C12	120.31 (8)	H18B—C18—H18C	109.5
O4—C10—C12	105.17 (7)	C11—C19—H19A	109.5
O3—C10—C12	93.54 (6)	C11—C19—H19B	109.5
N2—C11—O4	118.51 (8)	H19A—C19—H19B	109.5
N2—C11—C19	126.98 (9)	C11—C19—H19C	109.5
O4—C11—C19	114.51 (8)	H19A—C19—H19C	109.5
N2—C12—C13	113.66 (7)	H19B—C19—H19C	109.5
			
C2—N1—C1—O1	−156.91 (9)	C18—O5—C10—O3	−66.90 (11)
C17—N1—C1—O1	14.72 (14)	C18—O5—C10—C12	−176.84 (9)
C2—N1—C1—C9	29.21 (12)	C11—O4—C10—O5	136.31 (8)
C17—N1—C1—C9	−159.16 (8)	C11—O4—C10—O3	−95.49 (8)
C1—N1—C2—O2	175.59 (9)	C11—O4—C10—C12	4.29 (9)
C17—N1—C2—O2	3.89 (13)	C9—O3—C10—O5	−123.77 (8)
C1—N1—C2—C3	−1.26 (13)	C9—O3—C10—O4	109.48 (7)
C17—N1—C2—C3	−172.97 (8)	C9—O3—C10—C12	1.83 (7)
O2—C2—C3—C4	−10.44 (14)	C12—N2—C11—O4	2.05 (11)
N1—C2—C3—C4	166.32 (8)	C12—N2—C11—C19	−177.58 (9)
O2—C2—C3—C8	170.78 (9)	C10—O4—C11—N2	−4.29 (11)
N1—C2—C3—C8	−12.46 (13)	C10—O4—C11—C19	175.38 (8)
C8—C3—C4—C5	1.80 (14)	C11—N2—C12—C13	−136.26 (8)
C2—C3—C4—C5	−176.99 (9)	C11—N2—C12—C10	0.94 (10)
C3—C4—C5—C6	−2.53 (15)	C11—N2—C12—C9	89.12 (9)
C4—C5—C6—C7	0.91 (15)	O5—C10—C12—N2	−130.14 (9)
C5—C6—C7—C8	1.46 (14)	O4—C10—C12—N2	−3.31 (9)
C6—C7—C8—C3	−2.17 (13)	O3—C10—C12—N2	109.08 (7)
C6—C7—C8—C9	−178.83 (8)	O5—C10—C12—C13	1.60 (13)
C4—C3—C8—C7	0.55 (14)	O4—C10—C12—C13	128.44 (8)
C2—C3—C8—C7	179.30 (8)	O3—C10—C12—C13	−119.18 (8)
C4—C3—C8—C9	177.34 (8)	O5—C10—C12—C9	119.11 (9)
C2—C3—C8—C9	−3.91 (13)	O4—C10—C12—C9	−114.06 (7)
C10—O3—C9—C8	117.71 (8)	O3—C10—C12—C9	−1.67 (6)
C10—O3—C9—C1	−113.75 (8)	O3—C9—C12—N2	−101.08 (7)
C10—O3—C9—C12	−1.77 (6)	C8—C9—C12—N2	142.27 (8)
C7—C8—C9—O3	−24.24 (12)	C1—C9—C12—N2	12.45 (9)
C3—C8—C9—O3	159.04 (8)	O3—C9—C12—C13	125.77 (8)
C7—C8—C9—C1	−152.34 (8)	C8—C9—C12—C13	9.11 (11)
C3—C8—C9—C1	30.94 (11)	C1—C9—C12—C13	−120.70 (8)
C7—C8—C9—C12	79.06 (10)	O3—C9—C12—C10	1.65 (6)
C3—C8—C9—C12	−97.65 (10)	C8—C9—C12—C10	−115.00 (8)
O1—C1—C9—O3	14.35 (12)	C1—C9—C12—C10	115.18 (7)
N1—C1—C9—O3	−171.86 (7)	N2—C12—C13—C14	41.49 (11)
O1—C1—C9—C8	143.26 (9)	C10—C12—C13—C14	−86.40 (11)
N1—C1—C9—C8	−42.95 (10)	C9—C12—C13—C14	173.78 (7)
O1—C1—C9—C12	−84.84 (10)	C12—C13—C14—C15	64.00 (11)
N1—C1—C9—C12	88.95 (9)	C12—C13—C14—C16	−173.71 (8)
C18—O5—C10—O4	59.32 (11)		

### Hydrogen-bond geometry (Å, °)

**Table:**

*D*—H···*A*	*D*—H	H···*A*	*D*···*A*	*D*—H···*A*
C16—H16B···O5^i^	0.96	2.57	3.4213 (14)	148
C17—H17C···N2^ii^	0.96	2.59	3.5119 (14)	162

Symmetry codes: (i) −*x*+2, −*y*+2, −*z*+1; (ii) −*x*+2, −*y*+1, −*z*+1.
